# Axillary versus Forearm Crutches: A Prospective Cohort Comparing which is Superior for 3-Point Crutch Gait

**DOI:** 10.5704/MOJ.2107.006

**Published:** 2021-07

**Authors:** WMQ Yap, Z Hairodin, EBK Kwek

**Affiliations:** 1 Department of Orthopaedic Surgery, Tan Tock Seng Hospital, Singapore; 2 Department of Physiotherapy, Tan Tock Seng Hospital, Singapore; 3 Department of Orthopaedic Surgery, Woodlands Health Campus, Singapore

**Keywords:** crutch, gait, axillary crutch, forearm crutch, 3-point

## Abstract

**Introduction:**

Two common crutches utilised for orthopaedic rehabilitation include the axillary crutch and forearm crutch, with either crutch providing weight transfer through different mechanisms. This study aims to determine which crutch is best for patients, with specific reference to crutch gait and stability.

**Material and Methods:**

This is a level 2 prospective cohort study, recruiting 20 volunteers between 40 to 80 years old. Participants underwent 3 stations in 3 point crutch gait: straight line ambulation of 20m, timed-up-and-go-test, and computerised dynamic posturography. Participants also answered a subjective questionnaire on their crutch preferences.

**Results:**

Axillary crutches demonstrated a faster speed of ambulation compared to forearm crutches (Axillary crutch v=0.5m/s, Forearm crutch v=0.44m/s, p=0.002). There was a lower increase in heart rate post activity for axillary crutches. For the timed-up-and-go test, completing the circuit with Axillary crutches was faster (t=63.06, p<0.001) versus the forearm crutch (t=75.36, p<0.001). For computerised dynamic posturography, participants recorded lower effort scores for backward tilts when using axillary crutches (39.13, p=0.0497) versus forearm crutches (42.03, p=0.0497). Subjectively, majority of participants felt that axillary crutches had an easier learning curve and were superior in the areas of ambulation, balance and stability.

**Conclusion:**

Our study demonstrated that axillary crutches were superior to forearm crutches for 3-point crutch gait; axillary crutches had a faster ambulation speed, required less effort during use, provided superior stability and were the preferred choice subjectively. This study would be helpful for clinicians and therapists when prescribing mobility aids to individuals with impaired gait.

## Introduction

Crutches have always been a useful orthotic device to aid a patient’s gait, whether for post-operation rehabilitation or for strength training and muscle conditioning. The two most common types of crutches available at rehabilitation facilities and hospitals worldwide are axillary crutches and forearm crutches. Studies done comparing the 2 types of crutches are few and have shown varying results, with some concluding no difference, whilst some showing either crutch to be more effective. A paper published by Youdas *et al*^1^ in 2005 comparing partial weight bearing using conventional assistive devices concluded that both axillary and forearm crutches are equally effective in achieving partial weight bearing status. More recently, Goehring *et al*^2^ in 2011 concluded that with use of axillary crutches there was less postural sway and these crutches were perceived subjectively to be safer.

Earlier research focused on biomechanical studies^3^, while more recent research looked into varied parameters such as energy expenditure^4^ and postural sway^2^. These different clinical parameters used to measure crutch stability gave rise to differing results. To date, no studies have done a functional measurement of 3 point crutch gait. Three different systems have been described for underlying balance control in 3 point crutch gait: (i) Ambulatory speed, (ii) anticipatory postural adjustments prior to step initiation and sit-to-stand transition (time-up-and-go test), and (iii) dynamic postural stability. By having an objective and systemic evaluation of these systems, we hope to determine which type of crutch is best for patients with specific reference to 3 point crutch gait and crutch stability.

There is also paucity of literature on subjective patient reported scores of the different crutches. We also wanted to correlate if the objective scores reflected the same results to an individual’s subjective opinion.

## Materials and Methods

This was a level 2 prospective cohort study. The study was conducted at the physiotherapy unit of a tertiary care hospital. Twenty volunteers between the age of 40 years and 80 years old were selected from a larger pool of volunteers who had signed up for this study. The study was conducted over six months, and included the recruitment and tests that each participant was put through. Institutional ethics review board approval was obtained prior to the initiation of the study.

Inclusion criteria were healthy individuals with American Society of Anaesthesiologists (ASA) I or II physical status classification system, independent in their activities of daily living, and were able to ambulate 100m independently without assistance. Exclusion criteria were patients who had utilised crutches previously and those who had pre-existing upper or lower limb injuries, deformities or surgery that may result in a physical impairment to their ambulation. Pregnant females were also excluded from the study. Demographic data recorded included age, gender, body mass index (BMI) as well as racial group. Prior to the start of the study, participants were instructed on and practiced the correct method of using the axillary and forearm crutch for 3 point crutch gait (non-weight bearing on one affected leg throughout), supervised by either a doctor or a physiotherapist. Baseline heart rate, blood pressure, walking speed was measured.

**Fig. 1:**
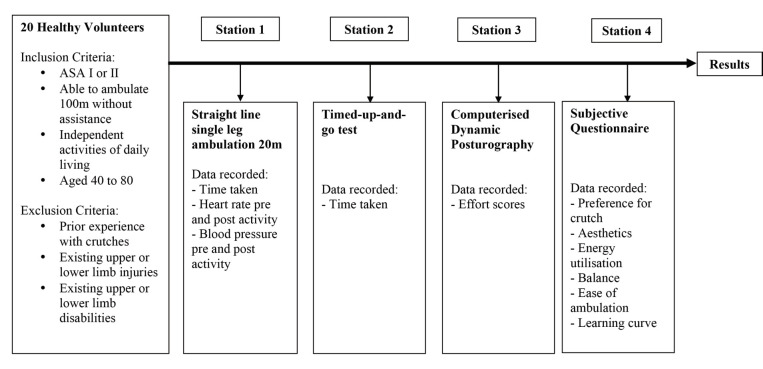
Study Protocol. The flowchart shows our study protocol from recruitment of patients through the 4 stations that each subject underwent. At each station, different parameters were recorded. Results were collated after the subject had completed all required stations.

All 20 participants were put through 3 stations and had to answer a questionnaire at the end of the study about their preference on which type of crutch was superior. Participants had to complete all 3 stations with both types of crutches while in 3 point crutch gait/stance. The order of the stations was fixed however crutch choice within each station was randomised. You may refer to (Fig. 1) for a diagram showing our study protocol. Short periods of rest were allowed (up to 30 minutes) within as well as between stations to allow the patient to recover fully to baseline before recording the next set of values. We foresaw that participant fatigability would be an important confounder in our results and this measure was adhered to strictly.

The 4 stations in the study are as follows: For station 1, participants were required to walk in a straight line distance of 20m in 3 point crutch gait. This was first done without any mobility aids, i.e. normal gait, recording time taken in seconds(s) by a stopwatch. Following that, a pair of crutches was selected at random and the same test was repeated for either crutch, with the participant non-weight bearing on one leg. The time taken for ambulation of 20m was recorded in seconds(s) by a stopwatch. Post ambulation heart rate and blood pressure were also recorded for either crutch.

In station 2, participants had to complete the timed-up-and-go test, which assesses a person’s mobility as well as static and dynamic balance over 20m. It is a test that is commonly used in rehabilitative assessment, with research showing excellent inter-rater and intra-rater reliability, correlating well with gait speed, Berg Balance scale and the Barthel Index^5,6^. This test comprises a participant starting in a seated position, getting up from his chair, putting on his/her crutches and getting into non-weight bear one leg position, before ambulating a distance of 10m (non-weight bearing one leg), circling around an obstacle and returning back to the start in a seated position. The time taken for the test is measured in seconds(s) by a stopwatch.

In station 3, participants were put through computerised dynamic posturography. This method has been validated by controlled research studies to isolate the functional contributions of somatosensory inputs and neuromuscular system ouputs for postural and balance control^7-9^. We utilised the Natus Medical NeuroComSmart Balance Master © machine for assessment. This machine utilises a 46 x 46cm dual force plate platform on which the patient stands while non-weight bearing on one leg. We utilised the exercise protocol “Adaptation test” which assesses a patient’s ability to minimise sway and regain balance when exposed to surface irregularities and unexpected changes in support surface inclinations. After the initial centre of gravity is identified, the force plate would tilt 5° forwards or backwards a total of 10 times. The participant is then required to maintain his balance on the force plate utilising his crutches to find his stable centre of gravity again. For each tilt, a numeric value is given that represents the sway energy (measured by the force exerted on the force plate and sway on the plate) exerted for recovery to equilibrium position. The readouts given by the machine are a numerical value (effort score) that quantify a calculation of the sway energy required to maintain balance when the platform tilted. A higher effort score represents a greater amount of energy/force exerted on the force plate to maintain balance, and implies the crutch is less stable. Results were recorded for both crutches in every participant, and the average sway energies compared between crutches.

At station 4, a subjective questionnaire was answered by the participants after the three tests had been conducted. Participants were asked to choose subjectively which crutch they felt was superior on the following parameters; learning curve, ease of ambulation, balance and stability, aesthetics and energy expenditure while ambulating. They were also asked which crutch they would purchase if they were injured and had to use one.

Statistical analysis was analysed using Stata 13 [StataCorp, College Station, TX]. Categorical variables were presented as numbers and percentage while continuous variable were presented as mean ± SD (standard deviation). Continuous variables were tested by paired t-test with two tailed significance level of <0.05 being used.

## Results

All 20 volunteers completed all stations required. The mean age of our patients was 48.85 with a range from 40 to 61 years old. Study population was almost evenly distributed with 11 males and 9 females. All of these patients were ASA I or II categories, with a mean body mass index (BMI) of 24.82. The baseline walking speed unassisted was 1.19m/s, which is comparable to most other biomechanical studies^10^ on gait analysis. The demographic information of our study population is summarised in (Table I).

**Table I:** Study population demographics

n=20 (mean ± SD)	
Age (years)	48.85 ± 6.95
Sex (n)	
Male	11
Female	9
BMI	24.82 ± 3.21
Mean Arterial Pressure (mmHg)	94.12 ± 8.78
Baseline walking speed (m/s)	1.19 ± 0.10

For station 1, there was a significant difference between forearm crutches and axillary crutches in terms of time taken to ambulate 20m, walking speed, and change in heart rate pre and post ambulation (Table II). Participants completed straight line ambulation of 20m with the axillary crutch (t=51.62s ± 27.55) about 8 seconds faster compared with when they used forearm crutches (t=59.39 ± 35.62). This result was statistically significant at p=0.04. Patients utilising axillary crutches were also able to walk faster (v=0.5m/s) compared with those in the forearm crutches (v=0.44m/s). This result was statistically significant at p=0.02. Pre and post ambulation heart rate (HR) increments were recorded, with the axillary crutch (HR Axillary=12.5 beats/min) demonstrating a lower heart rate increment post ambulation compared with forearm crutches (HR Forearm=15.85 beats/min). This result was statistically significant at p=0.030. The post ambulation mean arterial pressure (MAP) was not statistically significant (p=0.563), but this was higher for axillary crutches (MAP=3.23) compared with the forearm crutches (MAP= 2.1).

**Table II:** Station 1, Straight line ambulation 20m, non-weight bear one leg

	Forearm Crutch (mean ± SD)	Axillary Crutch (mean ± SD)	p value
Time taken (seconds)	59.39 ± 35.62	51.62 ± 27.55	0.040
Walking speed (m/s)	0.44 ± 0.22	0.5 ± 0.26	0.002
Change in heart rate	15.85 ± 11.08	12.5 ± 7.78	0.030
Increase in Mean arterial pressure post ambulation (mmHg)	2.1 ± 7.05	3.23 ± 9.46	0.563

For station 2, there was a significant difference between the two types of crutches for the timed up and go test (Table III). Patients ambulating with axillary crutches (t=75.36s ± 25.34) completed the circuit on average 12 seconds faster than those utilising forearm crutches (t=63.06s ± 19.23). This result was statistically significant at p<0.001.

**Table III:** Station 2, Timed-up-and-go test, non-weight bear single leg

	Forearm Crutch (mean ± SD)	Axillary Crutch (mean ± SD)	p value
Time taken to complete circuit (seconds)	75.36 ± 25.34	63.06 ± 19.23	<0.001

For station 3, in computerised dynamic posturography, the results are stratified into whether the force platform tilted forwards or backwards. A lower effort score translates to greater stability and balance. In the 5° backward tilt, the axillary crutch was the more stable crutch, with an average score of 39.13 for the axillary crutch versus 42.03 for the forearm crutch (Table IV). This result was statistically significant at p = 0.0497. The difference of score of 2.9 between the 2 crutches translated to a 6.9% increase in effort exerted when ambulating with the forearm crutch over the axillary crutch. In the 5° forward tilt, the axillary crutch showed marginally better results as well, with an average score of 32.15 against the forearm crutch score of 31.44 (Table IV). Though the forward tilt results were not significant (p=0.505), it serves as an indication on the more stable crutch.

**Table IV:** Station 3, Balance trainer, non-weight bear one leg

	Effort score Forearm Crutch (mean ± SD)	Effort score Axillary Crutch (mean ± SD)	p value
Tilt backward 5º average	42.03 ± 10.57	39.13 ± 9.16	0.0497
Tilt forward 5º average	32.15 ± 5.48	31.44 ± 5.75	0.505

**Fig. 2:**
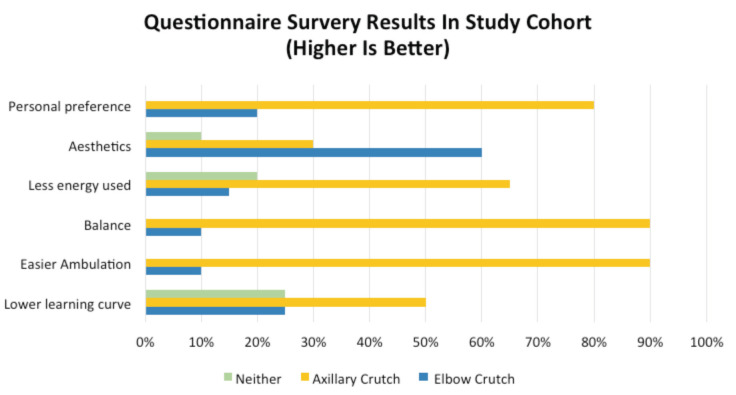
The bar chart shows individual responses to the questionnaire. The horizontal axis shows the percentages of people who chose either crutch. Participants were asked which crutch they preferred over in following domains (represented by vertical axis). Questionnaire results.

Results from the subjective questionnaire are represented in the bar chart (Fig. 2) attached. 80% of participants would choose an axillary crutch (80%) over a forearm crutch (20%) if they had to purchase one. A total of 90% of participants felt that the axillary crutch was superior to the forearm crutch in the areas of ambulation, and balance/stability. A total of 65% of participants also felt that the axillary crutch utilised less energy while ambulating. The results were equivocal for the learning curve of the crutches, being equivalent for both groups. In terms of aesthetics, participants preferred the design of the forearm crutch (60%) versus the axillary crutch (40%).

We also found that one-leg crutch ambulation would result in the walking speed of individuals being slowed by at least 50% compared to normal gait. The average normal walking speed of our study population was 1.19m/s ± 0.10 (comparable to the general population2). The average walking speed using axillary crutches was 0.5m/s ± 0.26, and walking speed using forearm crutches was 0.44 m/s ± 0.22.

## Discussion

With the 3 stations that each participant went through, we were able to objectively and systematically evaluate the three different systems underlying balance control in 3 point crutch gait: (i) Speed, (ii) anticipatory postural adjustments prior to step initiation and sit-to-stand transition, and (iii) dynamic postural stability. These three areas were sufficient for us to draw a conclusion that the axillary crutch was superior to the forearm crutch. This result differs from a prior study done by Youdas *et al*^1^ in 2005 that concluded that there was no difference between axillary and forearm crutches for partial weight bearing ambulation.

For the 20m straight line ambulation comparing the axillary and forearm crutches, the results conclusively demonstrate that axillary crutches have a faster speed as compared to forearm crutches. The axillary crutch design allows for a much more central anchor at the lateral chest level, providing additional truncal rotational stability^11,12^ as well as transferring the body weight through the stronger scapula and shoulder girdle muscles. The forearm crutch’s peripheral anchor transfers weight superiorly through to the forearm muscles and elbow joint^13^, which are more susceptible to rotational movements. We found that the additional stability provided by axillary crutches allowed the participants to have larger stride lengths and higher cadence, resulting in a faster speed. Results also showed that there was a higher change in heart rate post ambulation for forearm crutches against axillary crutches, telling us that participants put in a greater effort after ambulating with the forearm crutches as compared to the axillary crutches. This is likely attributed to lack of stability of the forearm crutches and a requirement for greater upper limb strength during their use.

The timed-up-and-go test is a cumulative assessment of many different parameters which include a patient’s sit-to-stand stability, ease of getting into single leg crutch stance, forward ambulation and turning balance^5^. The axillary crutch was faster than the forearm crutch by 12 seconds in this test. Participants using the forearm crutches were observed to slow down considerably while they were turning about an obstacle as they had to navigate a larger turning radius to maintain balance. This supports our earlier conclusion during straight line ambulation that forearm crutches are less stable.

For computerised dynamic posturography (CDP), previous studies have shown that an increase in CDP effort scores is directly related to higher risk of falls^14^. Both forward and backward tilt scores recorded a lower effort score when using axillary crutches versus forearm crutches. This result echoes findings from Goehring *et al*^2^, with axillary crutches showing a less postural sway versus forearm crutches. Findings for the CDP test differed depending on the direction of the force platform tilt. This was most likely due to the subjects’ crutch placement during the test, with majority of subjects using the crutch in a tripod position with the crutches slightly forward of the trunk. This resulted in a greater stability for forward tilts of the force platform, hence showing less difference on comparative data. Further studies are necessary for displacements of the body in all directions to determine if the results are also similar.

The significant difference in the subject’s perception of the axillary crutches being more stable during testing is consistent with the finding from our objective data. Given that the increase in CDP scores is related to falls^14^, this perception of safety reflects the additional stability provided by axillary crutches. Subjects also felt that the axillary crutch required less effort during use, and this corroborates our objective data showing a lower increase in heart rate post ambulation for axillary crutches.

Participants ambulating in 3 point crutch gait were noted to have reduced speed and increased effort, possible reasons include increased upper limb activity for utilisation of crutches, lack of familiarity with experimental equipment, and poorer balance and coordination in a one-leg stance. In addition, the researchers also observed the participants in 3 point crutch gait spent a longer amount of time in the stance phase, and a reduced time in swing phase.

This difference was exaggerated as the participants were nearing the end of their ambulation and increasingly fatigued. This leads us to believe that as the degree of disablement increases (either due to fatigue or physical impediment), the proportion of time spent in double crutch support increases and lower limb swing phase decreases.

As a patient progresses toward independent gait, the assistive devices they use should enable a patient to practice stability and balance with greater effort. One other walking aid used in earlier phases of rehabilitation is the assisted walker. Alkjaer *et al*^15^ in his analysis of the assisted walker showed that a large portion of the weight of the trunk was supported by the walker during ambulation, leading to reduced physical exertion and better stability. Our study shows that both forearm and axillary crutches have an increase in physical demands and are less stable than a walker, supporting its use in the middle phases of rehabilitation after the walker. We support findings from other studies that recommend a gradual progression in walking aids from assisted walker, thereafter followed by crutches, and subsequently followed by walking sticks/canes as a patient aims towards functional independence^16^, assessing a patient’s gait competency at each different phase of rehabilitation.

The relevance of this study is to better guide patients for rehabilitation options, as well as to provide institutional cost savings. The results from the different stations show that axillary crutches are superior to forearm crutches. This study had a number of limitations. Firstly, the sample size was small at 20 participants. Our study emphasis was placed on self-matching the participants and having a detailed study protocol with clear objective data. Having a larger sample size would yield more significant results, though from this sample size we can see a strong trend toward the superior crutch with the majority of the results showing significance. Secondly, our study focused only on two types of crutches. There are several newer crutch designs such as spring loaded crutches which were not assessed. The axillary and forearm crutches still remain by a large margin the two most common types of crutches available worldwide, owing to availability and cost. Further studies would need to be conducted to conclude if newer crutch designs were superior. Thirdly, our study was restricted to healthy subjects. The primary aim of the study was to directly compare two types of crutches, hence we avoided subjects with limb injuries as the varying levels of limb disability amongst subjects may introduce large confounders and variability in outcomes. It would be helpful to expand upon this study to evaluate the effect of different types of crutches on patients with specific limb disabilities.

## Conclusion

Our study demonstrated that axillary crutches were superior to forearm crutches for 3 point crutch gait; axillary crutches had a faster ambulation speed, required less effort during use and provided superior stability. On subjective questioning, our results are consistent with the objective data, with majority of participants preferring the axillary crutch over the forearm crutch for stability.
